# Infestation and Seasonal Fluctuation of Gamasid Mites (Parasitiformes: Gamasida) on Indochinese Forest Rat, *Rattus andamanensis* (Rodentia: Muridae) in Southern Yunnan of China

**DOI:** 10.3390/biology10121297

**Published:** 2021-12-08

**Authors:** Peng-Wu Yin, Xian-Guo Guo, Dao-Chao Jin, Wen-Yu Song, Lei Zhang, Cheng-Fu Zhao, Rong Fan, Zhi-Wei Zhang, Ke-Yu Mao

**Affiliations:** 1 The Provincial Key Laboratory for Agricultural Pest Management in Mountainous Region, Institute of Entomology, Guizhou University, Guiyang 550025, China; pwyin@dali.edu.cn (P.-W.Y.); jindaochao@163.com (D.-C.J.); 2 Vector Laboratory, Institute of Pathogens and Vectors, Yunnan Provincial Key Laboratory for Zoonosis Control and Prevention, Dali University, Dali 671000, China; merlin_song@hotmail.com (W.-Y.S.); leigezhang@163.com (L.Z.); zchengf2020@163.com (C.-F.Z.); fanfanlook1@outlook.com (R.F.); dalizzw@aliyun.com (Z.-W.Z.); 15887382535@163.com (K.-Y.M.)

**Keywords:** Acari, mite, ectoparasite, rodent, *Rattus*, small mammal

## Abstract

**Simple Summary:**

Gamasid mites are a large group of arthropods and some of them are ectoparasites on the body surface of rodents and some other small mammals. Some species of ectoparasitic gamasid mites are associated with the transmission of some zoonotic diseases such as rickettsialpox and hemorrhagic fever with renal syndrome (HFRS). Based on a 12-month consecutive investigation at Jingha village in southern Yunnan of China from April 2016 to March 2017, the present paper studied the infestation and seasonal fluctuation of gamasid mites on the Indochinese forest rat, *Rattus andamanensis*, the most dominant species of rodent at the investigated site. The temperature and rainfall are two key factors that influence the seasonal fluctuation of the mites.

**Abstract:**

A 12-month consecutive investigation was made at Jingha village in southern Yunnan of southwest China from April 2016 to March 2017. A total of 2053 Indochinese forest rats (*Rattus andamanensis* Blyth, 1860) were captured and examined, which account for 84.69% (2053/2424) of all the animal hosts (rodents and other small mammals) at the investigation site. And 39.82% (13,531/33,980) of gamasid mites were identified from the body surface of *R. andamanensis* and they belong to 41 species, 10 genera, 3 subfamilies and 2 families. Of the 41 species of gamasid mites identified from *R. andamanensis*, *Laelaps nuttalli* Hirst, 1915 and *Laelaps echidninus* Berlese, 1887 were the most dominant with 70.63% and 20.67% of constituent ratios respectively. In monthly fluctuations of all the gamasid mites on *R. andamanensis*, the constituent ratio (*C_r_*) and overall infestation mean abundance (*MA*) of the mites in 12 months showed two obvious peaks in January (winter season) and June (summer season). However, the two dominant mite species, *L. nuttalli* and *L. echidninus*, showed different patterns of seasonal fluctuations. *Laelaps nuttalli* occurred throughout the year, and its *C_r_* and *MA* showed two prominent peaks in winter season (December and January) and summer season (June), which belongs to the summer-winter type of seasonal fluctuation. *Laelaps echidninus* also occurred on *R. andamanensis* throughout the year, but its *C_r_* and *MA* showed only one peak in winter season (December and January), which belongs to the winter type of seasonal fluctuation. A negative correlation existed between two climatic factors (temperature and rainfall) and the infestations (*C_r_*, prevalence *P_M_* and *MA*) of two dominant mite species (*L. nuttalli* and *L. echidninus*) on *R. andamanensis* (*p* < 0.05). Temperature and rainfall are considered to be two key factors that influence the seasonal fluctuations of the mites on the studied rat species.

## 1. Introduction

Gamasid mites are a large group of arthropods with different ecological behaviors and they belong to the order Gamasida (or Mesostigmata) of the superorder Parasitiformes in the subclass Acari and the class Arachnoidea in zoological taxonomy [1,2]. The majority of gamasid mites are free living creatures and they can be found in the top soil, various humus and litter, animals nests, some stored goods or even on some plants [3,4]. Some gamasid mites are ectoparasites and they are often found on the body surface of rodents and some other small mammals [5,6]. The ectoparasitic gamasid mites can directly sting the human body and cause dermatitis, and some of them can be the vector or potential vector of some zoonotic diseases (zoonoses), such as rickettsialpox and hemorrhagic fever with renal syndrome (HFRS) [7,8]. Rodents and some other small mammals (insectivores and tree shrews) are the most important hosts of ectoparasitic gamasid mites [9,10,11,12].

As an important species of rodent, the Indochinese forest rat, *Rattus andamanensis* Blyth, 1860 has been documented in some regions of China, Vietnam, Laos, Cambodia, Thailand, central and northern Myanmar, northeastern India, Bhutan, eastern Nepal and Bangladesh [13]. In China, *R. andamanensis* is known to be mainly distributed in some regions of southern and southwestern China, such as Tibet, Guizhou, Hainan, Guangdong and Hong Kong [14]. A consecutive 12-month investigation for ectoparasitic gamasid mites was made at Jingha village (a localized site) in southern Yunnan of southwest China from April 2016 to March 2017, and *Rattus andamanensis* was found to be the most dominant species of small mammal hosts at the village. Based on the investigation data, the present paper analyzed the infestation and seasonal fluctuation of gamasid mites on *R. andamanensis* for the first time.

## 2. Materials and Methods

### 2.1. Field Investigation

**Figure 1:**
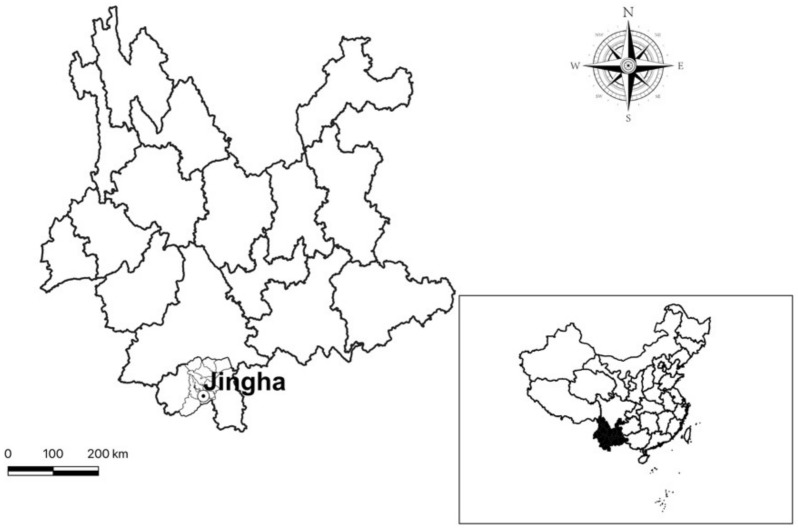
The locality of the field investigation site, Jingha village, Jinghong county, Xishuangbanna prefecture in the south of Yunnan province, China.

From April 2016 to March 2017, a consecutive 12-month field investigation was made at Jingha village, Jinghong county, Xishuangbanna prefecture in the south of Yunnan province (21°50′ N, 100°52′ E, 500–700 m a.s.l., Figure 1). Each month’s investigation lasted 15–20 days. Jingha village is a typical valley and flatland area near the coast of the Lancang River, a river from the northwest to the south in Yunnan province [15,16]. The village is a rubber planting area with lots of rubber woodlands dotted with some banana fields, farmlands, bush areas and broad-leaved forests. The meteorological data was from the online resources provided by the local weather forecasting department [17].

### 2.2. Collection and Identification of Gamasid Mites and Their Hosts

At the investigation site (Jingha village), the animal hosts of gamasid mites, rodents, and some other small mammals, were mainly captured with mousetraps (18 × 12 × 9 cm^3^, Guixi Mousetrap Apparatus Factory, Guixi, Jiangxi, China) every month. The mousetraps were set in the former evening and checked in the next morning. Each captured animal host was separately put in a white cloth bag and then brought to the field laboratory where the host was anesthetized with ether. Over a large white tray, the gamasid mites on the body surface of every host were collected with the help of a magnifier and the collected mites from each host were separately preserved in an Eppendorf tube containing 70% ethanol solution. After the mite collection, every animal host was identified to species according to its morphological features such as body size, body shape, body color and other measurements [13,14,18,19,20,21]. The identified *R. andamanensis* rats, together with the gamasid mites collected from the rats, were used as the target of the present paper. In the laboratory, the preserved gamasid mites were slide-mounted with Hoyer’s liquid which contains glycerin, the clearing reagent. The slide-mounted mite specimens were dried at 50 °C for about one week in an electric drying oven (DHG-9240A, Shanghai Yiheng Scientific Instrument Co., Ltd, Shanghai, China) through which the mounted mite specimens were gradually dehydrated and hyalinized. After dehydration and hyalinization, the mounted mite specimens were identified to species under the microscope (Nikon DS–Ri2, Nikon Corporation, Japan) with the help of relevant taxonomic literature including taxonomic monographs and identification keys [22,23,24,25,26]. The capture of animal hosts was officially approved by the local administration department of wildlife affairs. The ethical approval code or the permitted number was DLDXLL2020-1104. The representative specimens of gamasid mites and their animal hosts were deposited in the Institute of Pathogens and Vectors, Dali University, Dali, China.

### 2.3. Statistical Analysis

The constituent ratio (*C_r_*), prevalence (*P_M_*), mean abundance (*MA*) and mean intensity (*MI*) were used to calculate the infestation of *R. andamanensis* with gamasid mites, in which *C_r_* represents the percentage of a certain species of gamasid mite in all the mites, *P_M_* the percentage of the infested rat hosts with gamasid mites in all the rat hosts, *MA* the average number of gamasid mites per captured rat host and *MI* the average number of gamasid mites per infested rat host [7,27,28,29]. The richness index (*S*), Shannon-Wiener’s diversity index (*H′*), Pielou’s evenness (*E*) and Simpson’s dominance index (*D*) were used to calculate the community structure of the gamasid mite community on the rat *R. andamanensis* [27,30].

$$C_{r}=\frac{N_{i}}{N}\times 100\%;\;P_{M}=\left(\frac{H_{m}}{H}\right)\times 100\%;\;MA=\frac{N_{i}}{H};\;MI=\frac{N_{i}}{H_{m}};$$

$$S=\sum S_{i};\;H^{\prime }=-\overset{S}{\underset{I=1}{\sum }}\left(\frac{N_{i}}{N}\right)\ln\left(\frac{N_{i}}{N}\right);\;E=\frac{H^{\prime }}{\ln S};\;D=\overset{S}{\underset{i=1}{\sum }}{\left(\frac{N_{i}}{N}\right)}^{2}$$

In the above formulas, *N*i** represents the individuals of gamasid mite species *i. N* the total individuals of all the gamasid mites. *H* represents the total number of the rat hosts (*R. andamanensis*), *H_m_* the number of infested hosts by gamasid mite *m. S_i_* stands for gamasid mite species *i* in the gamasid mite community.

The correlation was analyzed between the seasonal fluctuation of dominant gamasid mites and the fluctuation of climatic factors and the significance level was at 0.05 (α = 0.05). Pearson correlation coefficient is calculated when the data conform to the normal distribution, and Spearman rank correlation coefficient is calculated when the data do not conform to the normal distribution.

## 3. Results

### 3.1. Collection of Gamasid Mites and Their Hosts

From the consecutive 12 months’ field investigation at Jingha village in southern Yunnan of China between April 2016 and March 2017, a total of 2,424 small mammal hosts were captured and they were identified into 15 species, 10 genera and 5 families under four orders, Rodentia, Erinaceomorpha, Soricomorpha and Scandetia. Of the identified 15 species of hosts, the abundance of *R. andamanensis* rats accounted for 84.69% (2053 rat individuals captured), which is the most dominant animal host at the investigation site, Jingha village. From the body surface of *R. andamanensis*, 13,662 gamasid mites were collected. The majority of the collected mites (13,531; 99.04%) were identified into 41 species, 10 genera, 3 subfamilies and 2 families (Table 1), and the remaining 131 mites have not been identified due to their body damage or ambiguous morphology.

### 3.2. Community Structure and Overall Infestations of Gamasid Mites on Rattus andamanensis

**Figure 2:**
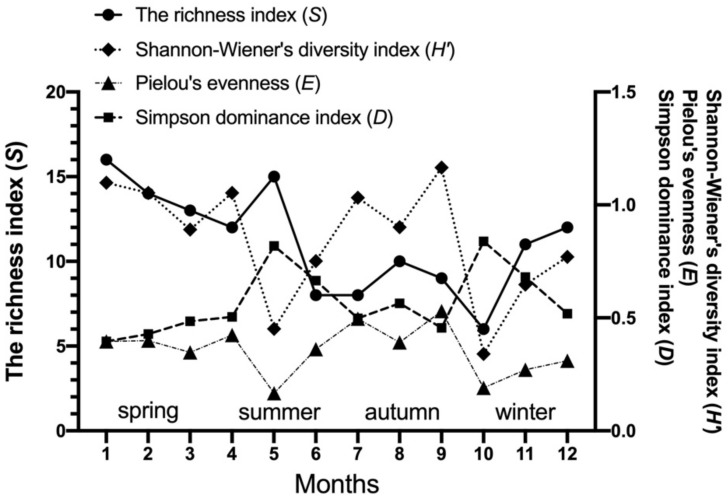
Monthly and seasonal fluctuation of community indices of gamasid mites on *Rattus andamanensis* at Jingha village in southern Yunnan of China (April 2016–March 2017).

**Figure 3:**
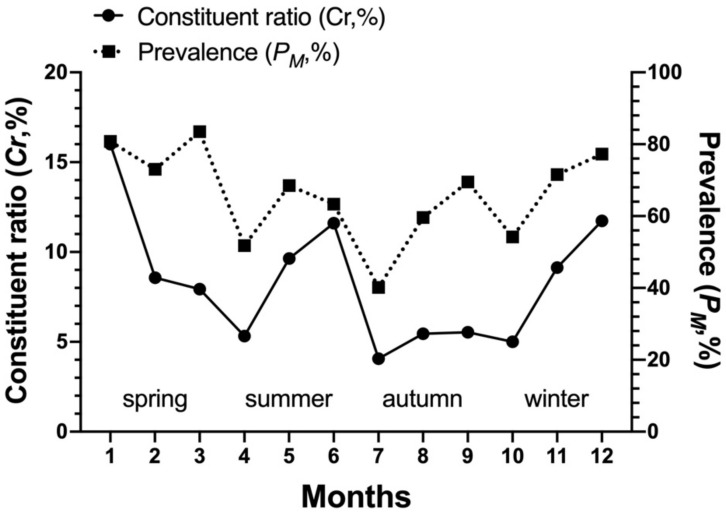
Monthly and seasonal fluctuation of overall constituent ratio (*C_r_*), prevalence (*P_M_*) of *Rattus andamanensis* with gamasid mites at Jingha village in southern Yunnan of China (April 2016–March 2017).

**Figure 4:**
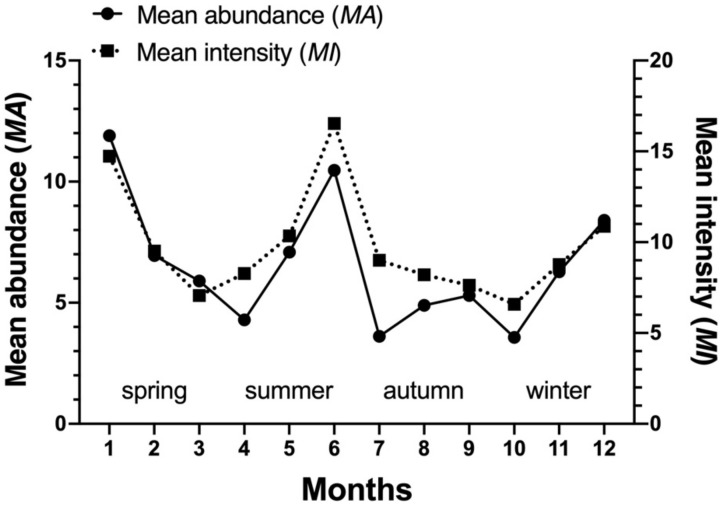
Monthly fluctuation of overall mean abundance (*MA*) and mean intensity (*MI*) of *Rattus andamanensis* with gamasid mites at Jingha village in southern Yunnan of China (April 2016–March 2017).

The community indices of gamasid mites on *Rattus andamanensis* fluctuated irregularly from month to month. The richness index (*S*) was the highest in January and the lowest in October, Shannon-Wiener’s diversity index (*H′*) was the highest in September and the lowest in October, Pielou’s evenness (*E*) was the highest in September and the lowest in May, and Simpson dominance index (*D*) was the highest in October and the lowest in January (Table 2, Figure 2). The constituent ratio (*C_r_*) and overall mean abundance (*MA*) of gamasid mites showed two obvious peaks in January (winter season) and June (summer season) (Table 3, Figure 3 and Figure 4).

### 3.3. Dominant Species of Gamasid Mites and Their Seasonal Fluctuations

The constituent ratios (*C_r_*) of *Laelaps nuttalli* (*C_r_* = 70.63%) and *L. echidninus* (*C_r_* = 20.67%) were the highest and they were the dominant species of gamasid mites on the studied rat species. In comparison with other species of gamasid mites, two dominant mite species showed relatively high prevalence (*P_M_*), mean abundance (*MA*) and mean intensity (*MI*) (Table 4). The monthly fluctuations of infestations of *R. andamanensis* with two dominant gamasid mite species were summarized in Table 5 and Table 6.

**Figure 5:**
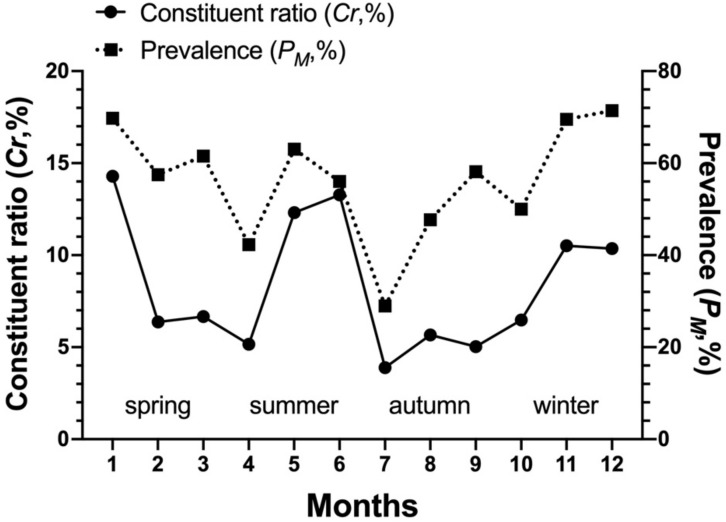
Monthly and seasonal fluctuations of constituent ratio (*C_r_*), prevalence (*P_M_*) of the gamasid mite *Laelaps nuttalli* on the rat host *Rattus andamanensis* at Jingha, southern Yunnan of China (April 2016–March 2017).

**Figure 6:**
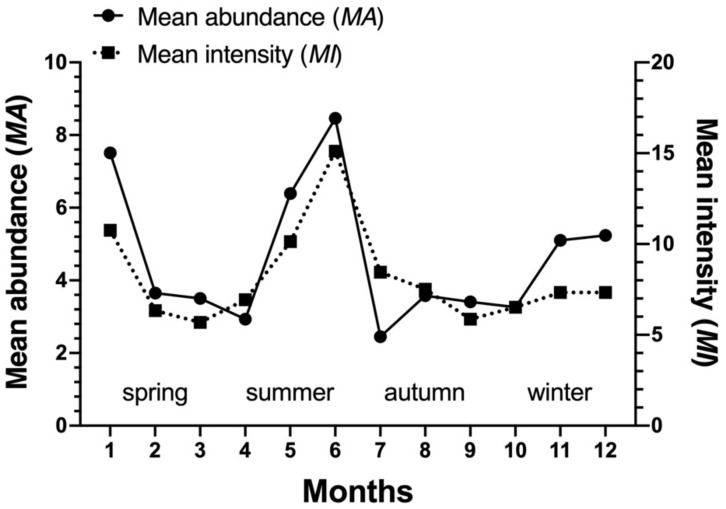
Monthly fluctuations of mean abundance (*MA*) and mean intensity (*MI*) of the gamasid mite *Laelaps nuttalli* on the rat host *Rattus andamanensis* at Jingha, southern Yunnan of China (April 2016–March 2017).

**Figure 7:**
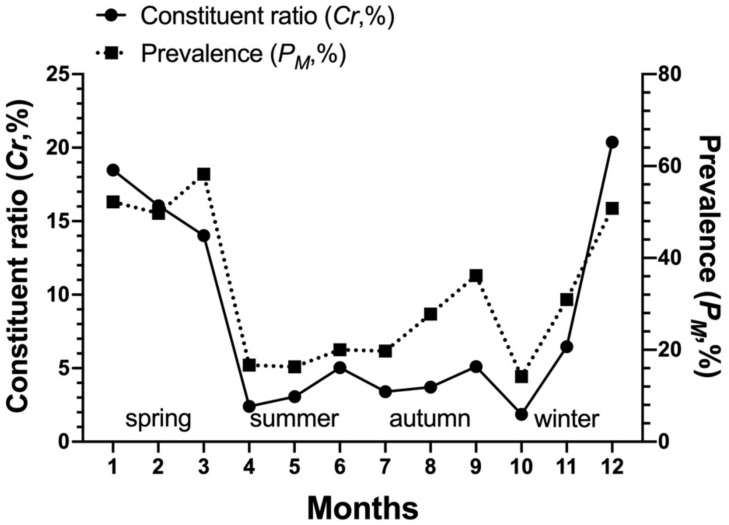
Monthly and seasonal fluctuations of constituent ratio (*C_r_*), prevalence (*P_M_*) of the gamasid mite *Laelaps echidninus* on the rat host *Rattus andamanensis* at Jingha, southern Yunnan of China (April 2016–March 2017).

**Figure 8:**
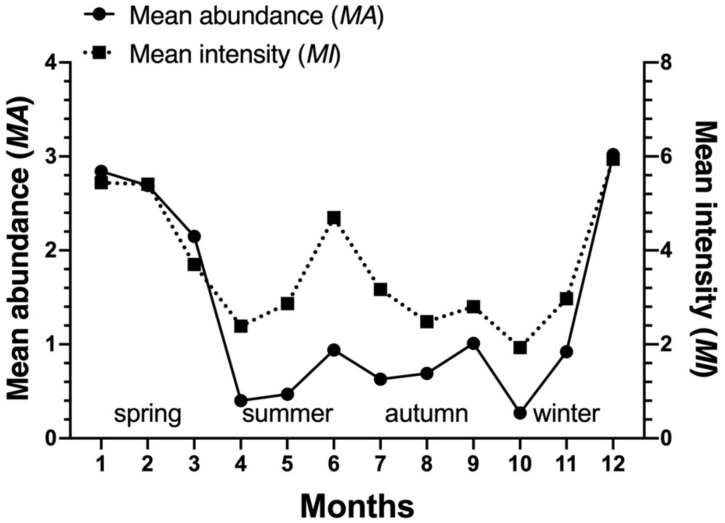
Monthly fluctuations of mean abundance (*MA*) and mean intensity (*MI*) of the gamasid mite *Laelaps echidninus* on the rat host *Rattus andamanensis* at Jingha, southern Yunnan of China (April 2016–March 2017).

*Laelaps nuttalli* occurred on the body surface of *R. andamanensis* throughout the year and its constituent ratio (*C_r_*) and mean abundance (*MA*) showed prominent peaks in winter season (December and January) and summer season (June) (Table 5, Figure 5 and Figure 6). *Laelaps echidninus* also occurred on *R. andamanensis* throughout the year and its *C_r_* and *MA* showed prominent peaks in winter season (December and January) (Table 6, Figure 7 and Figure 8).

### 3.4. Relationship between the Seasonal Fluctuation of Dominant Gamasid Mites and the Fluctuation of Climatic Factors

According to the meteorological data at the investigation site from April 2016 to March 2017, a correlation analysis was made between monthly climatic factors (temperature, rainfall and humidity) and monthly infestation indices (*C_r_*, *P_M_*, *MA* and *MI*) of gamasid mites on the rat host *R. andamanensis* using *R* statistical software (*R* version 3.5.3) [17]. The results showed that the rainfall was negatively correlated with the constituent ratio (*C_r_*) and prevalence (*P_M_*) of *L. echidninus* (*r* = −0.5874 for *C_r_* and *r* = −0.6224 for *P_M_*, *p* < 0.05). The average temperature was negatively correlated with the prevalence (*P_M_*) of *L. nuttalli* (*r* = −0.6922, *p* < 0.05). The temperature was negatively correlated with the constituent ratio (*C_r_*), prevalence (*P_M_*) and mean abundance (*MA*) of *L. echidninus* (*p* < 0.05). The correlation coefficient between the humidity and infestations of two dominant mite species, however, was of no statistical significance (Table 7).

## 4. Discussion

*Rattus andamanensis* was first named by Blyth in 1860, which was originally named *Mus (Leggada) andamanensis* Blyth (1860), and it was once described as a subspecies of *Rattus rattus* in some later literature [31]. Ellerman even thought that *R. andamanensis* was a synonym of *Rattus rattus brunneusculus* [32]. In some later literature, however, *R*. *r*. *brunneusculus* was described as an independent species, *R*. *brunneusculus* (the southeast Asian house rat) which was thought to be different from *R. andamanensis* [33]. Nowadays, much evidence supports that *R. andamanensis* is an independent species of rodent with a few confused synonyms such as *Mus burrulus*, *Mus flebilis*, *Epimys rattus klumensis*, *Epimys rattus kraensis*, *Rattus rattus koratensis*, *Rattus rattus hainanicus*, *Rattus confucianus yaoshanensis*, *Rattus rattus holchu*, *Rattus remotus*, *Rattus rattus brunneusculus* and *Rattus sikkimensis* [13,14,19,32,34,35,36,37,38,39]. In some literature published by Chinese scholars, however, *R. andamanensis* was often confused with *Rattus rattus sladeni*, *Rattus sladeni, Rattus brunneusculus* and *Rattus brunneusculus sladeni* [18,21,27,33,40,41,42,43].

Previously, Lv et al. reported the infestation and seasonal fluctuation of chigger mites at Jingha village of southern Yunnan, the same localized area with the present paper. Influenced by the above -mentioned confusion, the Indochinese forest rats (*R. andamanensis*) were incorrectly identified as the southeast Asian house rats (*R. brunneusculus*) in this report [44]. After Lv et al.’s publication, we reexamined the rat specimens repeatedly and found that they are actually Indochinese forest rats (*R. andamanensis*), and the southeast Asian house rat (*R. brunneusculus*) was misused in the above-mentioned paper [44]. Therefore, here we use the correct name (*R. andamanensis*) instead of the previously misused identification.

The results of the present study showed that the captured *R. andamanensis* rats accounted for 84.69% (2053/2424) of all the 15 species of animal hosts at the investigation site (Jingha village in southern Yunnan of China), which were the most dominant host species at the village. The abundant *R. andamanensis* rats may be associated with the special habitat of the investigation site. As a typical wild rodent species, *R. andamanensis* often lives in various wild habitats such as forests, bush areas, and farmlands [14,19]. The investigation site, Jingha village, is a rubber planting area with lots of rubber woodlands dotted with some banana fields, farmlands, bush areas and broad-leaved forests, and this kind of wild habitat seems to be suitable for the survival and reproduction of *R. andamanensis*.

Similar to other species of rodents, *R. andamanensis* is an important host for some ectoparasites such as chigger mites, gamasid mites, fleas and sucking lice [31]. The identified 41 species of gamasid mites from such a single rodent species (*R. andamanensis*) at such a confined local area (Jingha village) even exceed the total number of gamasid mite species reported in other provinces or local regions of China. According to previous reports, 8 gamasid mite species were reported in Taiwan, 11 species in Dezhou of Shandong province, 13 species in Sandu’ao Island of Fujian province and 13 species in the northeastern border of China (four cities together) [45,46,47,48]. The number of Gamasida species in the present study also exceeds the number of gamasid mite species in some reports from other countries. For example, 8 species were recorded in Kuala Lumpur and the states of Selangor and Negeri Sembilan, and 10 species at Ulu Muda Forest Reserve, in Kedah of Malaysia [49,50]. The abundant species of gamasid mites on *R. andamanensis* at Jingha village reflect a high species richness and species diversity of the mites on a single rodent species at a localized area. The result suggests that *R. andamanensis* in southern Yunnan of China has a high potential to harbor lots of gamasid mite species.

As two dominant species of gamasid mites found on the studied rat host, *L. nuttalli* (*C_r_* = 70.63%) and *L. echidninus* (*C_r_* = 20.67%) had a relatively high prevalence (*P_M_*), mean abundance (*MA*) and mean intensity (*MI*) (Table 4). *Laelaps nuttalli* and *L. echidninus* are two common species of gamasid mites found on the body surface of many animal hosts and the hosts’ nests with low host specificity. Some previous studies showed that *L. nuttalli* and *L. echidninus* often occurred on the body surface of some rat species in the genus *Rattus* simultaneously and they were often the dominant species of gamasid mites [51,52,53]. The result of the present study is consistent with the previous studies, and it implies that *L. nuttalli* and *L. echidninus* may have some preference to *Rattus* rats [51,52,53]. As the world-distributed species of gamasid mites, *L. nuttalli* and *L. echidninus* are widely distributed in Asia, Europe, America, Africa, Australia, and other parts of the world [1,46,47,49,50,51,54,55,56,57,58,59,60], and they often co-exist on the same species of hosts in the same geographical regions [61,62,63,64,65,66,67,68]. From north to south, *L. nuttalli* and *L. echidninus* have been recorded in many parts of China such as Heilongjiang, Jilin, Jiangsu, Hunan, Hubei, Guangdong, Guangxi, Fujian, Hainan, Sichuan, Chongqing, Guizhou, Yunnan, Taiwan, and Hong Kong, etc. To date, *L. nuttalli* and *L. echidninus* have been found on the body surface of more than 30 host species which involves rodents, insectivores, pikas and tree shrews, and some rat species of the genus *Rattus*, e.g., *R. tanezumi* Temminck, 1844, *R. norvegicus* Berkenhout, 1769, *R. nitidus* Hodgson, 1845, *R. losea* Swinhoe, 1871 and *R. andamanensis*, are the most common hosts [2,6,8,69,70]. *Laelaps nuttalli* and *L. echidninus* can invade human beings to cause skin irritation and dermatitis [71]. Moreover, *L. echidninus* is suspected of being associated with some zoonoses such as Q fever, rickettsialpox and pseudotuberculosis, etc., for the related pathogens were once isolated from it [72]. The frequent occurrence of *L. echidninus* with a high constituent ratio on some *Rattus* rats including *R. andamanensis* may have some potential significance of preserving or transmitting the causative agents of some zoonotic diseases.

The present paper described the seasonal fluctuations of *L. nuttalli* and *L. echidninus* on *R. andamanensis* for the first time. The two principal mite group showed different patterns of seasonal fluctuations. *Laelaps nuttalli* mites occurred throughout the year, and its constituent ratio (*C_r_*) and mean abundance (*MA*) showed two prominent peaks in winter season (December and January) and summer season (June), which belongs to the summer-winter type of seasonal fluctuation (Figure 5 and Figure 6). *Laelaps echidninus* mites also occurred throughout the year, but its *C_r_* and *MA* showed only one peak in winter season (December and January), which belongs to the winter type of seasonal fluctuation (Figure 7 and Figure 8). The seasonal fluctuations of *L. nuttalli* and *L. echidninus* on *R. andamanensis* in southern Yunnan of China seem to be different from those of the same mite species on some other rat hosts and in some other geographical regions. In the border areas of northeast China, the seasonal fluctuations of *L. nuttalli* on *R. norvegicus* rats showed two peaks in May and October [46]. In Taiwan, the highest seasonal peak of *L. nuttalli* and *L. echidninus* on *R. losea* rats was in autumn [48]. In Argentina, *L. echidninus* had the highest infestation frequency on *R. norvegicus* in spring [58]. The above results suggest that the seasonal fluctuations of *L. nuttalli* and *L. echidninus* may vary with different species of hosts and different geographical regions. At present, it is difficult to explain why *L. nuttalli* and *L. echidninus* showed different seasonal fluctuation patterns, and further studies are needed. 

The previous reports demonstrated that temperature and rainfall are two key factors which influence the seasonal fluctuation of chigger mites [44]. In the present study, a negative correlation existed between two climatic factors (temperature and rainfall) and the infestations (*C_r_*, *P_M_* and *MA*) of two dominant mite species (*L. nuttalli* and *L. echidninus*) on *R. andamanensis* rats (*p* < 0.05), which suggests that temperature and rainfall are two key factors that influence the seasonal fluctuations of the gamasid mites, *L. nuttalli* and *L. echidninus*. However, we cannot speculate how the temperature and rainfall influence the seasonal fluctuations of *L. nuttalli* and *L. echidninus* and their dynamic variations, and more studies are still needed in the future. 

## 5. Conclusions

The Indochinese forest rat (*R. andamanensis*) in southern Yunnan of China has a high potential to harbor a lot of gamasid mite species. *Laelaps nuttalli* and *L. echidninus* are the dominant species of Gamasida on *R. andamanensis* and they have different patterns of seasonal fluctuations. The seasonal fluctuation of *L. nuttalli* belongs to the summer-winter type, and that of *L. echidninus* belongs to the winter type. Temperature and rainfall (precipitation) are two key factors which influence the seasonal fluctuation of gamasid mites.

## Figures and Tables

**Table 1:** Systematic list of gamasid mites from the body surface of Indochinese forest rats (*Rattus andamanensis*) at Jingha village in southern Yunnan of China (April 2016–March 2017).

Taxa	N	Taxa	N	Taxa	N	Taxa	N
Family Lealapidae	13,530	*L. jettmari* Vitzthum, 1930	1	Genus *Cosmolaelaps*	6	Subfamily Haemogamasinae	11
Subfamily Lealapinae	13,410	*L. extremi* Zachvatkin, 1948	3	*C. yerulyuae* Ma, 1995	6	Genus *Eulaelaps*	9
Genus *Laelaps*	13,337	Genus *Androlaelaps*	4	Subfamily Hypoaspidinae	109	*E. jilinensis* Wen, 1976	2
*L. nuttalli* Hirst, 1915	9557	*A. singularis* Wang *et* Li, 1965	4	Genus *Hypoaspis*	109	*E. pratentis* Zhou, 1981	1
*L. liui* Wang et Li, 1965	104	Genus *Tricholaelaps*	15	*H. concinna* Teng, 1982	21	*E. substabularis* Yang *et* Gu, 1986	1
*L. guizhouensis* Gu *et* Wang, 1981	34	*T. myonysognathus* Grochovskaya *et* Nguen-Xuan-hoe, 1961	15	*H. aculeifer* Canestrini, 1884	16	*E. stabularis* Koch, 1836	5
*L. echidninus* Berlese, 1887	2797	Genus *Dipolaelaps*	26	*H. chelaris* Teng, Zhang *et* Cui, 1992	16	Genus *Haemogamasus*	2
*L. turkestanicus* Lange, 1955	216	*D. jiangkouensis* Gu, 1985	25	*H. ovatus* Ma, Ning *et* Wei, 2003	8	*H. monticola* Wang *et* Li, 1965	1
*L. traubi* Domrow, 1962	219	*D. chimmarogalis* Gu, 1983	1	*H. lubrica* Voigts *et* Oudemans, 1904	6	*H. nidi* Michael, 1892	1
*L. fukienensis* Wang, 1963	110	Genus *Haemolaelaps*	22	*H. pavlovskii* Bregetova, 1956	35		
*L. algericus* Hirst, 1925	84	*H. casalis* Berlese, 1887	4	*H. kirinensis* Chang, Cheng *et* Yin, 1963	3	Family Macronyssidae	1
*L. cheni* Li, 1965	116	*H. orientalis* Teng *et* Pan, 1964	13	*H. leeae* Tseng, 1977	2	Genus *Ornithonyssus*	1
*L. jinghaensis* sp.nov	22	*H. cordatus* Teng *et* Pan, 1964	3	*H. hrdyi* Samsinak, 1961	1	*O. bacoti* Hirst, 1913	1
*L. chin* Wang *et* Li, 1965	29	*H. glasgowi* Ewing, 1925	1	*H. linteyini* Samsinak, 1964	1		
*L. clethsionomydis* Lange, 1955	45	*H. petauristae* Gu *et* Wang, 1980	1				

**Table 2:** Monthly fluctuation of community indices of gamasid mites on *Rattus andamanensis* at Jingha village in southern Yunnan of China (April 2016–March 2017).

Years and Months		Community Structure of Gamasid Mites			
Years	Months	*S*	*H’*	*E*	*D*
2017	1	16	1.098	0.396	0.396
	2	14	1.053	0.399	0.428
	3	13	0.891	0.347	0.485
2016	4	12	1.053	0.424	0.504
	5	15	0.451	0.166	0.818
	6	8	0.751	0.361	0.665
	7	8	1.032	0.496	0.498
	8	10	0.901	0.391	0.564
	9	9	1.165	0.530	0.456
	10	6	0.340	0.190	0.839
	11	11	0.647	0.270	0.681
	12	12	0.770	0.310	0.518
Total		41	1.013	0.273	0.542

Annotation: The field investigation at Jingha village was made between April 2016 and March 2017, which forms a consecutive process from January to December.

**Table 3:** Monthly fluctuation of overall infestations of *Rattus andamanensis* with gamasid mites at Jingha village in southern Yunnan of China (April 2016–March 2017).

Months	Host (*R. andamanensis*)		Mites		Host Infestation		
	N	*C_r_* (%)	No. of Mites	*C_r_* (%)	*P_M_*	*MA*	*MI*
					(%)		
1	182	8.87	2165	16.00	80.77	11.90	14.73
2	167	8.13	1160	8.57	73.05	6.95	9.51
3	182	8.87	1074	7.94	83.52	5.90	7.07
4	168	8.18	720	5.32	51.79	4.29	8.28
5	184	8.96	1304	9.64	68.48	7.09	10.35
6	150	7.31	1570	11.60	63.33	10.47	16.53
7	152	7.40	549	4.06	40.13	3.61	9.00
8	151	7.36	738	5.45	59.60	4.89	8.20
9	141	6.87	748	5.53	69.50	5.30	7.63
10	190	9.25	678	5.01	54.21	3.57	6.58
11	197	9.60	1237	9.14	71.57	6.28	8.77
12	189	9.21	1588	11.74	77.25	8.40	10.88
Total	2053	100.00	13,531	100.00	66.63	6.59	9.89

**Table 4:** Constituent ratios and infestations of two dominant species of gamasid mites (*Laelaps nuttalli* and *Laelaps echidninus*) on the rat host *Rattus andamanensis* at Jingha village in southern Yunnan of China (April 2016–March 2017).

Dominant Mite Species	Mites		Host Infestation		
	N	*C_r_* (%)	*P_M_* (%)	*MA*	*MI*
*L. nuttalli*	9557	70.63	57.04	4.66	8.16
*L. echidninus*	2797	20.67	33.07	1.36	4.12
Total	12,699	91.30			

**Table 5:** Monthly fluctuations of constituent ratios and infestations of the gamasid mite *Laelaps nuttalli* on the rat host *Rattus andamanensis* at Jingha village in southern Yunnan of China (April 2016–March 2017).

Months	Examined Hosts	Infested Hosts	No. of Mites	*C_r_* (%)	*P_M_* (%)	*MA*	*MI*
1	182	127	1366	14.29	69.78	7.51	10.76
2	167	96	609	6.37	57.49	3.65	6.34
3	182	112	637	6.67	61.54	3.50	5.69
4	168	71	493	5.16	42.26	2.93	6.94
5	184	116	1176	12.31	63.04	6.39	10.14
6	150	84	1269	13.28	56.00	8.46	15.11
7	152	44	372	3.89	28.95	2.45	8.45
8	151	72	541	5.66	47.68	3.58	7.51
9	141	82	481	5.03	58.16	3.41	5.87
10	190	95	619	6.48	50.00	3.26	6.52
11	197	137	1004	10.51	69.54	5.10	7.33
12	189	135	990	10.36	71.43	5.24	7.33
Total	2053	1171	9557	100.00	57.04	4.66	8.16

**Table 6:** Monthly fluctuations of constituent ratios and infestations of the gamasid mite *Laelaps echidninus* on the rat host *Rattus andamanensis* at Jingha village in southern Yunnan of China (April 2016–March 2017).

Months	Examined Hosts	Infested Hosts	No. of Mites	*C_r_* (%)	*P_M_* (%)	*MA*	*MI*
1	182	95	517	18.48	52.20	2.84	5.44
2	167	83	449	16.05	49.70	2.69	5.41
3	182	106	392	14.02	58.24	2.15	3.70
4	168	28	67	2.40	16.67	0.40	2.39
5	184	30	86	3.07	16.30	0.47	2.87
6	150	30	141	5.04	20.00	0.94	4.70
7	152	30	95	3.40	19.74	0.63	3.17
8	151	42	104	3.72	27.81	0.69	2.48
9	141	51	143	5.11	36.17	1.01	2.80
10	190	27	52	1.86	14.21	0.27	1.93
11	197	61	181	6.47	30.96	0.92	2.97
12	189	96	570	20.38	50.79	3.02	5.94
Total	2053	679	2797	100.00	33.07	1.36	4.12

**Table 7:** Pearson correlation between two dominant species of gamasid mites and climatic factors (total rainfall, average temperature and average humidity) at Jingha village in southern Yunnan of China (April 2016–March 2017).

Species of Gamasid Mites	Infestation Indices	Pearson Correlation, *r* (*p*)		
		Total Rainfall (mm)	Average Temperature (°C)	Average Humidity (%)
*L. nuttalli*	*C_r_*	0.0946 (0.77)	−0.2698 (0.3963)	0.1023(0.7517)
	*P_M_*	−0.5441 (0.0674)	−0.6922 (0.0126) *	−0.0211(0.948)
	*MA*	0.035 (0.9212)	−0.3627 (0.2466)	0.1761 (0.5841)
	*MI*	0.3706 (0.2367)	0.1761 (0.5841)	0.2747 (0.3876)
*L. echidninus*	*C_r_*	−0.5874 (0.0488) *	−0.6373 (0.0258) *	−0.1408 (0.6624)
	*P_M_*	−0.6224 (0.0348) *	−0.6444 (0.0237) *	−0.1408 (0.6624)
	*MA*	−0.5734 (0.0555)	−0.6549 (0.0208) *	−0.0845 (0.794)
	*MI*	−0.3279 (0.2981)	−0.3591 (0.2516)	−0.2113 (0.5098)

Annotation: The figures in the Table represent the coefficients of Pearson’s correlation (*r*), and the figures in the brackets stand for the probability of significance (*p*). The “*” represents statistically significant (double tail, α = 0.05).

## Data Availability

The experimental data used to support the findings of this study are available from the corresponding author request.
